# Optimization of the genotyping‐by‐sequencing SNP calling for diversity analysis in cape gooseberry (*Physalis peruviana* L.) and related taxa

**DOI:** 10.1371/journal.pone.0238383

**Published:** 2020-08-26

**Authors:** Felix E. Enciso-Rodríguez, Jaime A. Osorio-Guarín, Gina A. Garzón-Martínez, Paola Delgadillo-Duran, Luz Stella Barrero

**Affiliations:** Centro de Investigación Tibaitatá, Corporación Colombiana de Investigación Agropecuaria–Agrosavia, Mosquera, Cundinamarca, Colombia; University of Delhi, INDIA

## Abstract

A robust Genotyping-By-Sequencing (GBS) pipeline platform was examined to provide accurate discovery of Single Nucleotide Polymorphisms (SNPs) in a cape gooseberry (*Physalis peruviana* L.) and related taxa germplasm collection. A total of 176 accessions representing, wild, weedy, and commercial cultivars as well as related taxa from the Colombian germplasm bank and other world repositories were screened using GBS. The pipeline parameters mnLCov of 0.5 and a mnScov of 0.7, tomato and potato genomes, and cape gooseberry transcriptome for read alignments, were selected to better assess diversity and population structure in cape gooseberry and related taxa. A total of 7,425 SNPs, derived from *P*. *peruviana* common tags (unique 64 bp sequences shared between selected species), were used. Within *P*. *peruviana*, five subpopulations with a high genetic diversity and allele fixation (H_E_: 0.35 to 0.36 and F_IS_: -0.11 to -0.01, respectively) were detected. Conversely, low genetic differentiation (F_ST_: 0.01 to 0.05) was also observed, indicating a high gene flow among subpopulations. These results contribute to the establishment of adequate conservation and breeding strategies for Cape gooseberry and closely related *Physalis* species.

## Introduction

Cape gooseberry (*Physalis peruviana* L.) is an herbaceous Solanaceae species, native to the Andean region, with enormous potential for biomedical research and commercial purposes [1]. The mating system of cape gooseberry is mostly outcrossed that, along with the occurrence of mixed-ploidy, indicates a transitioning state from wild to cultivated [2, 3], possibly due to a lack of a domestication process as has occurred in major crops of the Solanaceae family such as tomato or potato [4, 5]. Globally, this berry-bearing species is known for its nutritional value, possessing high contents of vitamins and minerals, as well as anti-inflammatory, antioxidant and disease control (diabetes and hypertension) properties [6–10]. These characteristics have enabled its distribution as a crop to other parts of the world, including Africa, Asia, and Oceania [11, 12]. Colombia is the world’s leading producer of this exotic fruit, with roughly 16,109 metric tons produced in 2018 [13], followed by Ecuador, Zimbabwe, Malaysia, China, Kenya, and South Africa [14].

Cape gooseberry production in the Andean region has been diminished due to important phytosanitary problems caused mainly by bacterial (*Xanthomonas* sp., and *Ralstonia solanacearum*), Oomycete (*Phytium* sp.), and fungal pathogens (*Alternaria* sp., *Cladosporium* sp., *Cercospora* sp., *Phoma* sp., *Sclerotinia sclerotiorum*, *Fusarium oxysporum*, among others) [14, 15]. Among them, *F*. *oxysporum* sp. physali (*Foph*) [16], represents one of the most damaging diseases. Therefore, leveraging favorable allelic combinations from wild, weedy, landraces and related taxa into cultivated cape gooseberry populations will contribute to reducing production losses and environmental impacts; since, to date, the only way to manage these pathogens is through chemical control.

The available genetic diversity of cultivated and wild related taxa has helped to establish appropriate conservation, management, and sustainable utilization strategies of different crops [17]. In particular, for cape gooseberry and related taxa, the proper characterization of available germplasm collections will contribute to the identification of resistant or tolerant sources to biotic and abiotic stresses. Not only will this help with battling pathogens, but will increasingly assist with future challenges due to climate change [18].

Molecular markers have become valuable tools to assess genetic variation of worldwide plant repositories. Population studies, marker assisted selection, mapping and association studies, among other applications, have been used for this purpose [19]. To date, some studies have been conducted to characterize the genetic diversity of cape gooseberry. Random amplified microsatellites (RAM) markers have been used by two independent studies on panels of 43 and 18 accessions respectively. They detected a low differentiation and high heterozygosity levels among the majority of the accessions [20, 21]. Other studies used more informative markers, such as simple sequences repeats (SSRs), conserved ortholog sequences II (COSII), immunity related genes (IRGs) and single nucleotide polymorphisms (SNPs) to analyze the diversity levels and population structure in natural and breeding *P*. *peruviana* populations [22–26]. However, these low-throughput platforms provide a limited ability to estimate the extent of cape gooseberry genetic variability, since they focus on limited genomic regions.

Recently, next generation sequencing (NGS) technologies have accelerated the screening of germplasm collections, identifying thousands of SNP markers in a cost effective and timely way [27, 28]. Notably, genotyping-by-sequencing (GBS), a highly multiplexed method based on the reduction of genome complexity through methylation-sensitive restriction enzymes [29], has become a popular approach for detecting genome-wide variation in plants [30]. Previous studies in cape gooseberry have identified about 50,000 SNPs using GBS in 100 accessions from the Colombian germplasm collection, for association studies related to fruit quality and *Foph* resistance [31, 32].

Although these previous advances in genetic diversity and association mapping have contributed to the exploration of cape gooseberry genetic resources, the species still lacks a more comprehensive GBS-SNP pipeline platform for SNP discovery. This study mainly seeks to provide a robust GBS-SNP calling pipeline for this species and related taxa by obtaining common genomic regions between tomato, potato, and cape gooseberry using the previously developed TasselPipelineGBS [33]. Moreover, it aims to provide an extensive study of a larger germplasm collection using GBS, which comprises 158 accessions of *P*. *peruviana* and 18 wild related species from the *Physalis* genus, including 95 technical replicates, to leverage the genetic diversity and population structure of this Andean crop towards conservation and sustainable utilization strategies.

## Materials and methods

### Plant material and DNA isolation

One hundred and fifty-eight cape gooseberry (*Physalis peruviana* L.) and 18 related taxa accessions, containing one to seven individuals each, for a total of 644 individuals, were used in this study (Table 1). This germplasm collection is maintained by the Colombian Corporation for Agricultural Research (AGROSAVIA). These accessions, collected mainly across the Colombian Andean mountains, were selected based largely on geographic distribution and state of cultivation (S1 Table).

**Table 1:** Cape gooseberry and related taxa accessions used in this study.

Species	Number of accessions	Number of individuals
*Physalis peruviana*	158	587
*Physalis angulata*	2	9
*Physalis floridana*	2	8
*Physalis ixocarpa*	1	2
*Physalis philadelphica*	10	34
*Physalis pruinosa*	1	1
*Physalis viscosa*	1	1
*Physalis* sp.	1	2
**Total**	**176**	**644**

The genomic DNA was isolated from young leaves using the DNeasy Plant Mini Kit (QIAGEN, Germany) following the manufacturer's procedure. DNA quantity was determined using NanoDrop (Thermo Scientific® ND 2000) and λ DNA/HindIII Ladder (Promega, Madison, USA). DNA quality was assessed using EcoRI restriction enzyme digestions (New England Biolabs, Beverly, MA) and visualized on 1% (w/v) agarose gels stained with ethidium bromide (0.5 μg/mL).

### Genotyping and read alignment

GBS library generation and Illumina sequencing were conducted at the Institute for Genomic Diversity (IGD) from Cornell University (Ithaca, New York, USA). An additional 95 individuals previously sequenced [31] were included as technical replicates in this study (S1 Table). FASTQ files containing 739 individuals (644 plus 95 technical replicates) were processed using the GBS pipeline implemented on Tassel standalone V4.3.5 [34]. Since cape gooseberry does not have a reference genome, we used the closely related sequenced species tomato (*Solanum lycopersicum*) and potato (*Solanum tuberosum*) [35, 36] as well as the cape gooseberry leaf transcriptome (SRA: SRP005904) [37] for reads alignment and later SNP discovery.

### Parameter selection for SNP calling

Different GBS parameters in the DiscoverySNPCaller and GBSHapMapFilters plugins were used for SNP calling and hapmap filtering, respectively (Table 2), using a minimum read depth of 5. Additionally, homologue genomic regions among tomato and potato genomes, and cape gooseberry transcriptome were used. This was done to avoid possible bias caused by copy number variations and ploidy complexity during SNP calling. This approach was performed by selecting common tags (a unique sequence of 64 bp in length, excluding the barcode and shared among the selected species) using the TagsOnPhysicalMap (TOPM) file. Finally, to further reduce marker redundancy, the high linkage disequilibrium filter was implemented.

**Table 2:** GBS parameters used for SNP calling and Hapmap filtering in this study.

Plugin*	Parameter	Parameter Abbreviation	Threshold Values	Description
DiscoverySNPCaller	Minimum locus coverage	mnLCov	0.1, 0.5, and 0.7	Uses the proportion of individuals with at least one tag present from the TagLocus covering a SNP
	Minimum minor allele count	mnMAC	20	Selects SNPs that pass the specified mnMAC
	Average sequencing error per base	errRate	0.05	Decides between heterozygous and homozygous calls
	Minimum minor allele frequency	mnMAF	0.05	Selects SNPs that pass the specified mnMAF
GBSHapMapFilters	Minimum site coverage	mnScov	0.1, 0.5, and 0.7	Uses the minimum taxon call rate for a SNP to be included in the output where taxon call rate is the proportion of the taxa with individuals that are not missing for that SNP
	Minimum taxon coverage	mnTCov	0.5	Uses the minimum taxon call rate for a SNP to be included in the output where call rate is the proportion of the SNP individuals for a taxon that are not missing.

* Information gathered from: https://bytebucket.org/tasseladmin/tassel-5-source/wiki/docs/TasselPipelineGBS.pdf

### Cluster and principal component analyses

The clustering patterns of the 739 individuals were compared through genetic distances generated by Tassel V4.3.5 in order to verify which parameters were correctly assessed. The parameter selection was based on genetic trees in which most of the technical replicates clustered together with their corresponding counterpart and those which better grouped the accessions according to their passport data (S1 Table). A total of 54 different genetic trees were generated based on the neighbor-joining (NJ) method [38], using filtered SNPs derived from datasets with or without common tags. From the selected SNP data set, a principal component analysis (PCA) was performed, in which each SNP marker was scored with 0 for the homozygous allele aa, 1 for the heterozygous allele Aa and 2 for the homozygous allele AA, based on the allele counting for each marker. The newly generated matrices were subsequently analyzed using the gdsfmt and SNPRelate packages implemented on the statistical software R [39].

### Population structure and genetic diversity analyses

The population structure and genetic diversity analyses were carried out only for *P*. *peruviana* accessions using the best GBS parameter combinations selected from the NJ analysis. The software ADMIXTURE V.1.23 [40], was used to estimate the individual’s genetic ancestry, calculating the optimum subpopulation (K) number, by the use of cross-validation tests ranging from K = 1 to K = 10.

Genetic diversity, referred to as expected heterozygosity (H_E_), was calculated as $1-\sum_{i=1}^{k}p_{i}^{2}$, where ***p_i_*** is the frequency of the ***i^th^*** allele for ***k*** alleles. Observed heterozygosity (H_O_) and H_E_ for each locus were estimated using Genepop version 4.7.5 [41]. Finally, to estimate the overall genetic divergence among subpopulations within the cape gooseberry germplasm collection, the genetic differentiation (F_ST_) and fixation (F_IS_) indices were calculated using the software mentioned above.

## Results

### SNP calling and dataset selection

Two major categories were used for SNP calling, using independent assemblies with the tomato and potato genomes, and the cape gooseberry transcriptome, based on whether or not common tags were used. For those SNP calls derived without common tags, around 9 million tags were generated. Between 91,692 and 6,189 SNPs within each reference genome/transcriptome were identified (Table 3) with an average depth of 6.3. As expected, with less stringent parameters (mnLCov = 0.1, and mnScov = 0.1), the percentage of missing data was higher, ranging from 44.31 to 48.27%. With more stringent parameters (mnLCov = 0.7, and mnScov = 0.7), the percentage of missing data was reduced by a factor of 10 (4.36 to 4.47%, Table 3). Conversely, the number of heterozygous SNPs was lower, ranging from 16.13% (low parameter values) to 45.57% (high parameter values, Table 3).

**Table 3:** SNPs identified for cape gooseberry and related taxa using different parameters in the standalone script of Tassel. Two reference genomes (tomato, potato) and a transcriptome (cape gooseberry) were used for SNP calling.

Parameters			Without common tags			With common tags		
			*S*. *lycopersicum*	*S*. *tuberosum*	*P*. *peruviana*	*S*. *lycopersicum*	*S*. *tuberosum*	*P*. *peruviana*
mnLCov 0.1	mnScov 0.1	SNPs	83,792	91,692	52,179	16,986	16,552	15,772
		Miss^†^	48.17	48.27	44.31	42.81	43.20	41.65
		Het^§^	16.13	16.20	17.03	17.70	18.01	18.51
	mnScov 0.5	SNPs	40,101	43,411	29,339	9,637	9,370	9,393
		Miss	18.35	18.11	18.29	16.90	16.88	16.86
		Het	32.06	32.42	29.26	29.84	30.94	30.20
	mnScov 0.7	SNPs	28,969	31,765	21,994	7,632	7,324	7,459
		Miss	9.91	9.96	11.14	10.67	10.49	10.78
		Het	40.29	40.31	35.53	34.89	36.55	35.20
mnLCov0.5	mnScov 0.1	SNPs	40,567	44,304	30,649	9,666	9,649	9,591
		Miss	21.05	21.11	21.20	19.60	19.63	19.56
		Het	26.14	26.21	26.36	28.27	27.92	28.57
	mnScov 0.5	SNPs	39,581	43,117	29,666	9,372	9,369	9,312
		Miss	19.25	19.23	19.01	17.21	17.40	17.28
		Het	26.85	26.92	27.28	29.28	28.81	29.48
	mnScov 0.7	SNPs	29,257	32,068	22,302	7,573	7,431	**7,425**^ß^
		Miss	12.46	12.63	12.63	11.97	11.98	12.08
		Het	32.45	32.40	32.61	33.38	33.39	33.91
mnLCov0.7	mnScov 0.1	SNPs	8,952	9,213	6,549	2,397	2,475	2,443
		Miss	7.22	7.62	8.47	8.55	8.7	8.20
		Het	44.05	43.42	42.51	41.90	41.71	42.41
	mnScov 0.5	SNPs	8,695	8,921	6,238	2,318	2,365	2,361
		Miss	4.7	4.83	4.73	5.03	5.17	4.75
		Het	45.48	44.92	44.65	43.88	43.73	44.23
	mnScov 0.7	SNPs	8,602	8,809	6,189	2,285	2,328	2,331
		Miss	4.36	4.39	4.47	4.63	4.74	4.45
		Het	45.57	45.08	44.72	44	43.90	44.27

^†^ Percentage of missing data.

^§^ Percentage of heterozygosity.

^ß^ SNP number selected for subsequent analysis, according to the best parameter combination.

**Fig 1:**
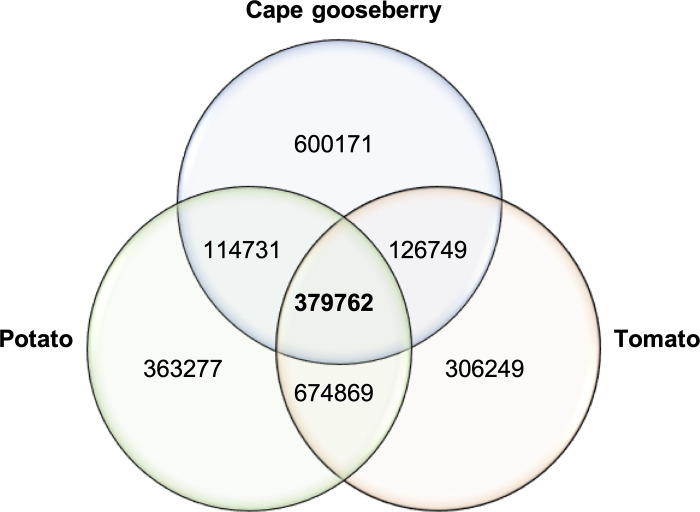
Venn diagram showing tags in common between the tomato, potato and cape gooseberry obtained after read alignment using the GBS pipeline. A common tag refers to a 64 bp read-length that is shared among the three selected species.

With common tags, low tag numbers were obtained between tomato, potato and cape gooseberry (379,762), after the Illumina reads alignment (Fig 1). These common tags were later used for SNP calling using the GBS pipeline with the parameters described herein. The SNPs identified with common tags, ranged from 2,285 to 16,986, for high and low parameter values respectively. The percentage of missing data ranged between 4.45 and 43.2%, and an observed heterozygosity between 17.7 and 44.27% (Table 3), for high and low parameter values, respectively.

### Cluster and PCA analyses for cape gooseberry and related taxa

A total of 54 genetic trees were generated, corresponding to each parameter and whether or not common tags were evaluated (Table 3), using the 95 technical replicates and their counterparts within the initial 644 selected individuals. The parameter arrangement with a mnLCov of 0.5 and a mnScov of 0.7 showed the closest genetic distance of all the trees generated, with approximately 70% of the technical replicates grouping in the same cluster as their counterparts. From this parameter combination, a total of 7,425 high quality SNPs (resulting from common tags for *P*. *peruviana* transcriptome) were selected to evaluate the clustering pattern in cape gooseberry and related taxa.

**Fig 2:**
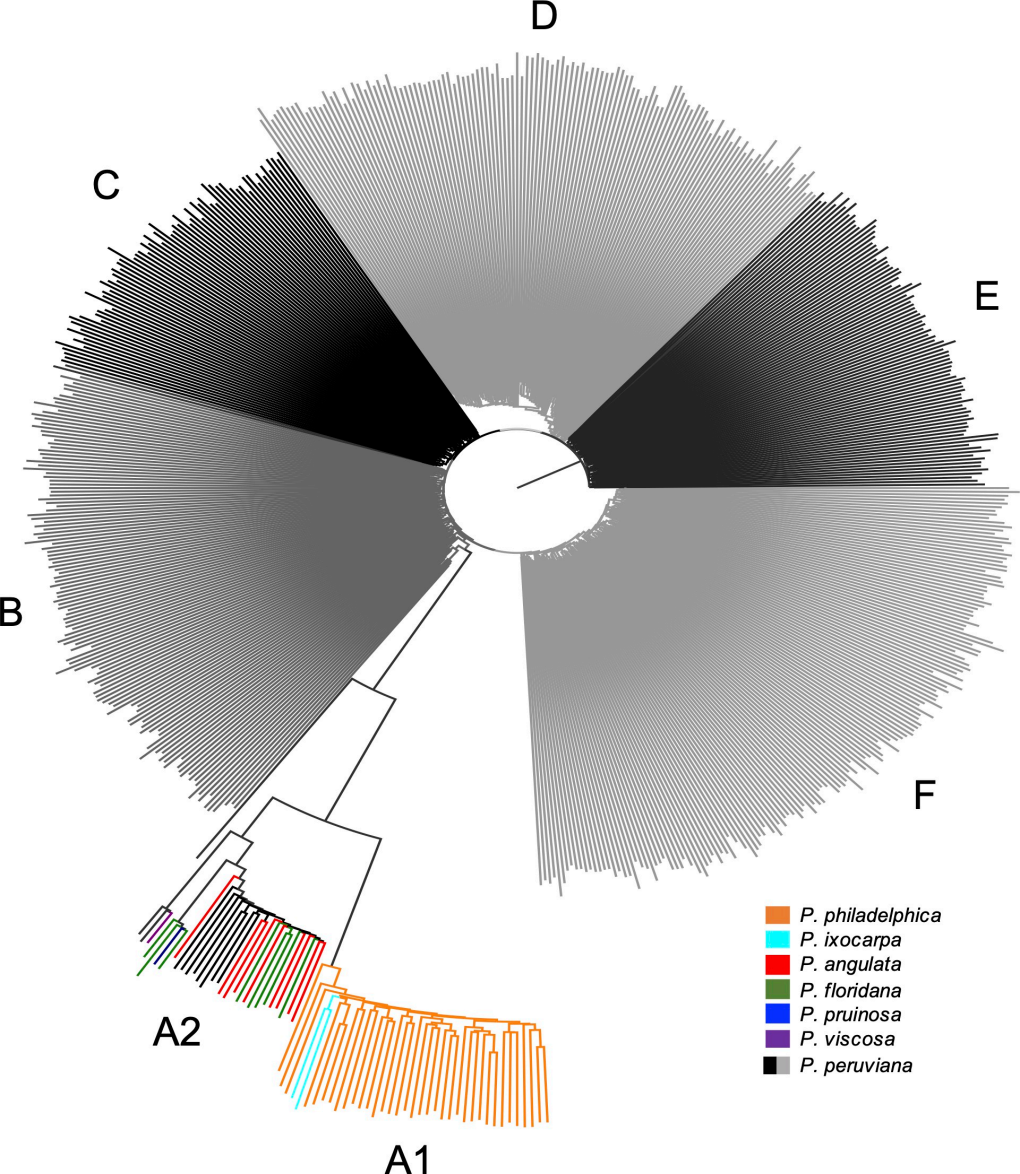
Neighbor-joining (NJ) tree for cape gooseberry and related taxa generated using 7,425 SNPs derived from cape gooseberry common tags. Groups A1 and A2 represent related taxa clusters, and B-F represent five sub-populations within the *P*. *peruviana* population. Each branch represents an individual plant.

The best NJ tree includes six clusters in which clear patterns emerged for the population under study (Fig 2). Cluster A includes two sub-groups comprised of all related taxa. Specifically, group A1 harbors all *P*. *philadelphica* accessions and one accession from *P*. *ixocarpa*, and A2 harbors two accessions from both *P*. *angulata*, and *P*. *floridana*, and one accession from both *P*. *pruinosa* and *P*. *viscosa*. We also observed three *P*. *peruviana* accessions (09U207, 09U289 and 09U291) in group A2. Furthermore, most cape gooseberry accessions (including cultivated, weedy, and wild), clustered in five different groups. Clusters B, C, D and E concentrated most accessions from the cape gooseberry producing regions, while cluster F harbors a high number of wild accessions from the Antioquia and Nariño departments of Colombia.

**Fig 3:**
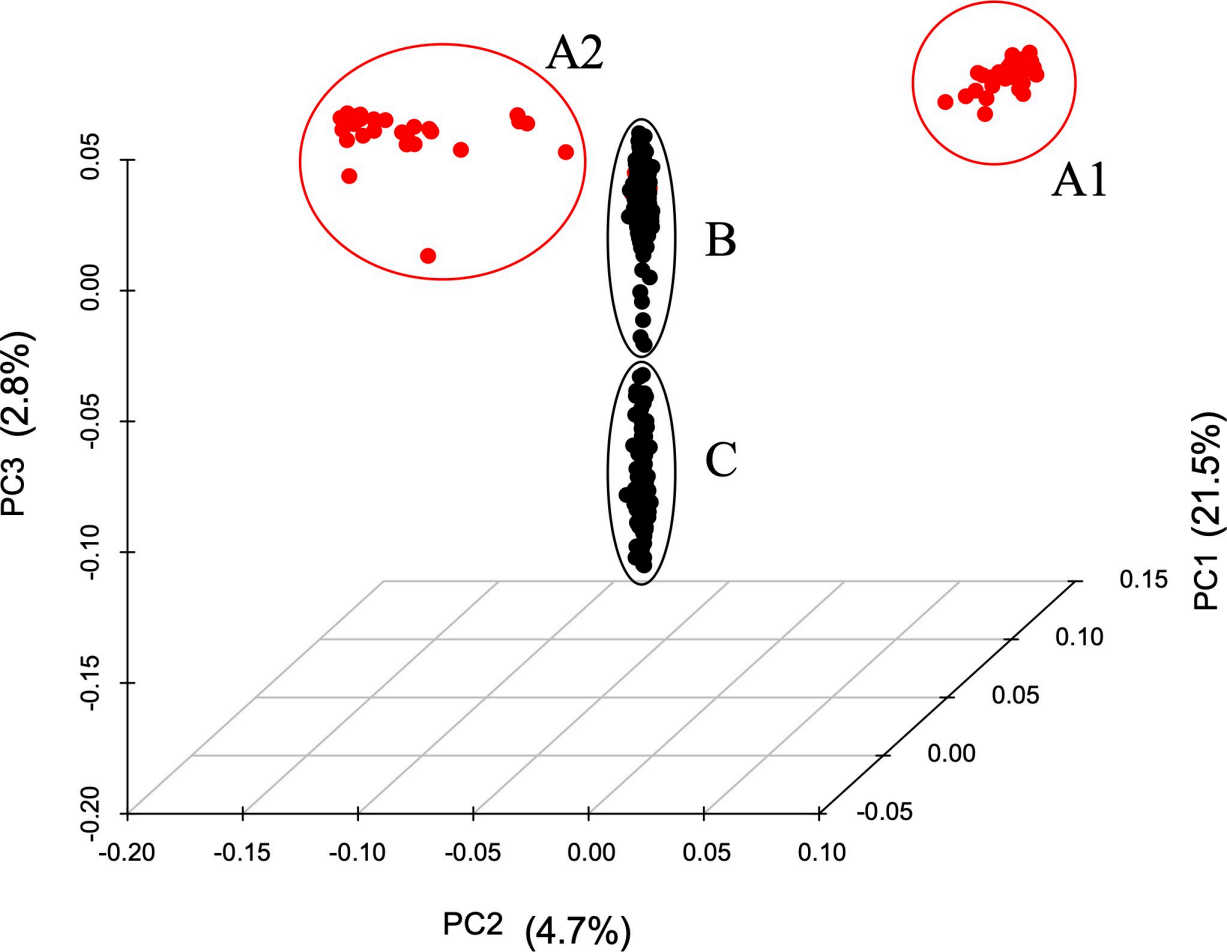
Population structure of cape gooseberry and realated taxa as revealed by a Principal Component Analysis (PCA). The first three components uncovered four clusters. Most *P*. *peruviana* accessions grouped in two clusters at the main centroid (B and C, black dots), and the related taxa were located in two separate clusters (A1 and A2, red dots).

Moreover, the PCA revealed that the first three principal components explained 29% of the total variance, in which the first component contributed for almost all the variance observed, with 21.5%, followed by the second and third component with 4.7% and 2.8%, respectively (Fig 3).

In Fig 3, each cluster represents a distinctive cape gooseberry and related taxa grouping pattern. Clusters B and C include most of the cultivated accessions coming from the main cape gooseberry producing regions in Colombia (Boyacá, Cundinamarca, Antioquia and Nariño departments). In contrast, clusters A1 and A2 contain mostly related taxa. Specifically, group A1 harbors all *P*. *philadelphica* accessions and one accession from *P*. *ixocarpa*, while Group A2 includes *P*. *angulata*, *P*. *floridana*, *P*. *pruinosa* and *P*. *viscosa* as well as three *P*. *peruviana* accessions (09U207, 09U289 and 09U291, S1 Table) as observed in the NJ tree.

### Population structure and genetic diversity in cape gooseberry

**Fig 4:**
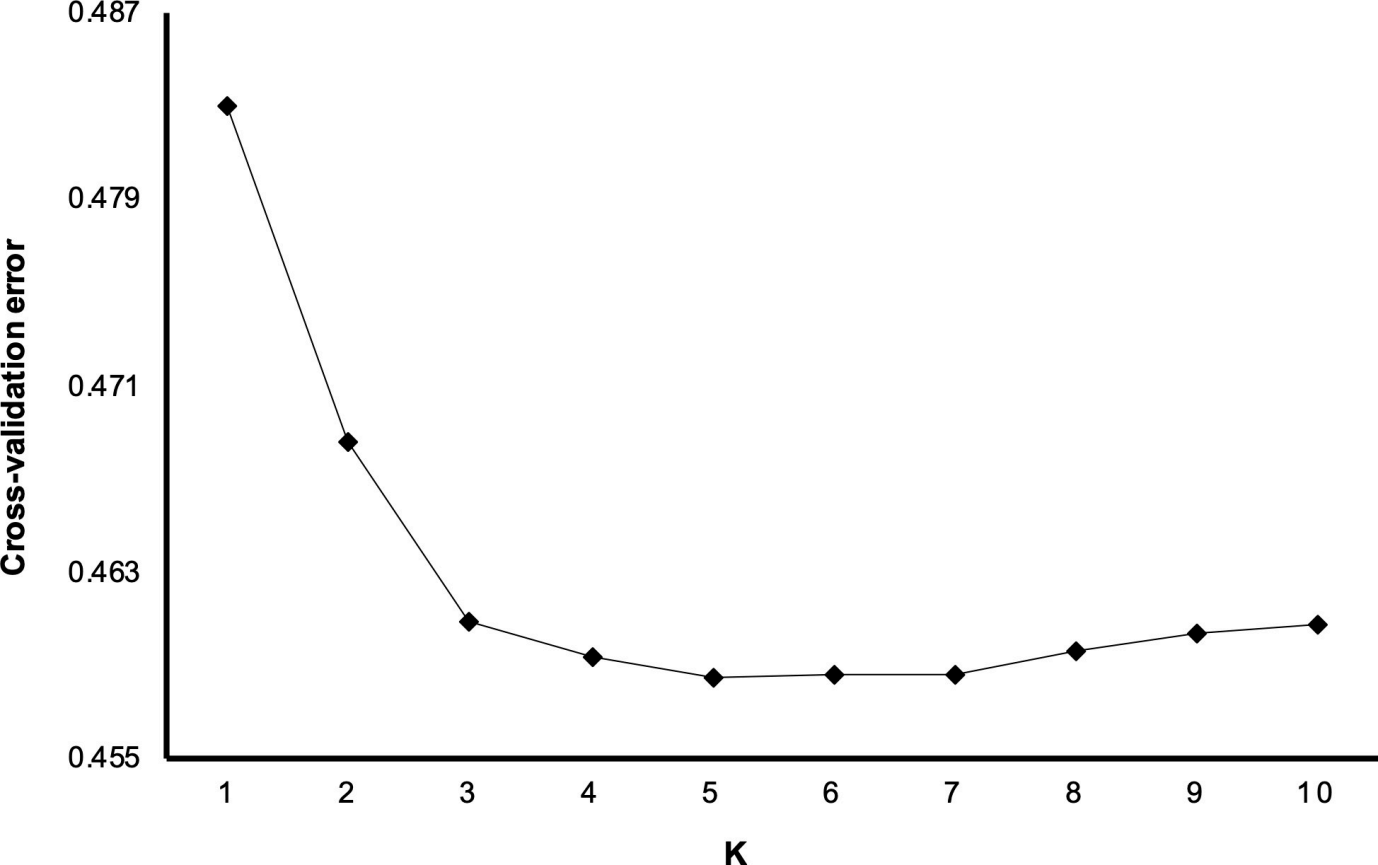
Cross validation plot for subpopulation (K) estimation in cape gooseberry. The smallest cross validation error value was observed when K = 5.

**Fig 5:**
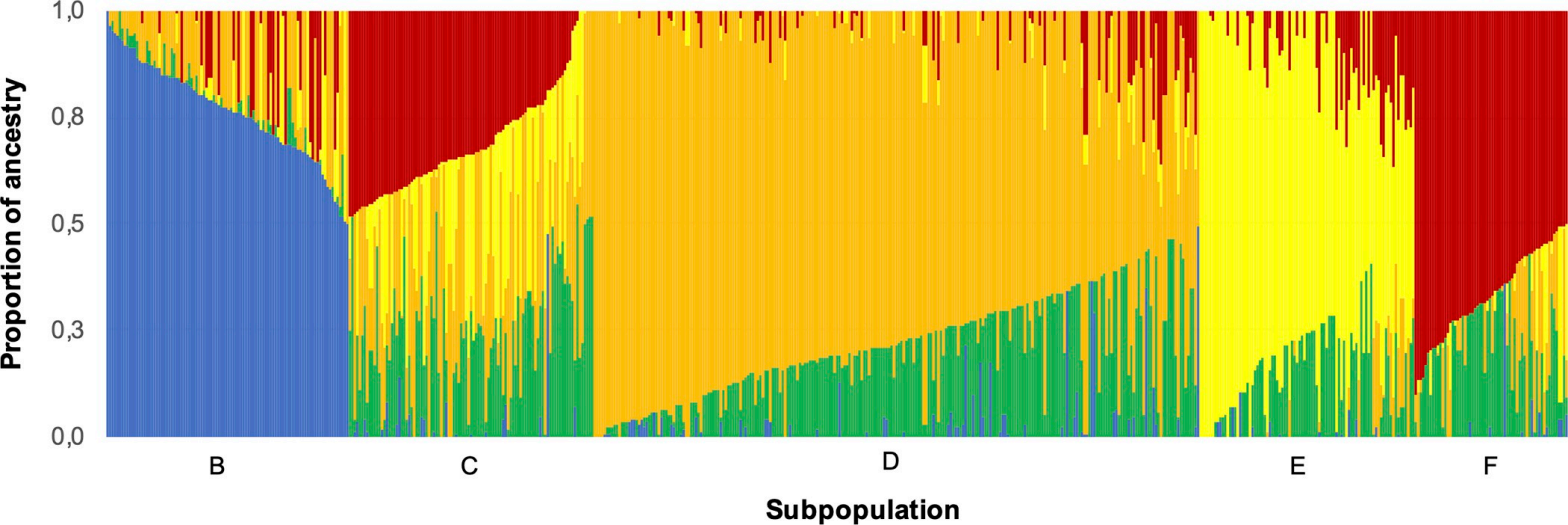
Population stratification of *P*. *peruviana* as revealed by ADMIXTURE. Five subpopulations were classified based on ancestry values.

To further analyze the population structure and genetic diversity within *P peruviana*, the related taxa and *P*. *peruviana* accessions clustering in sub-groups A1 and A2 in the NJ and PCA analyses were removed. The cross-validation procedure implemented in ADMIXTURE enabled the identification of five subpopulations within *P*. *peruviana* (K = 5, Fig 4), in accordance to the number of clusters shown in the NJ tree for this species (Fig 2). In Fig 5, subpopulations B and D include accessions mostly from the Colombian departments with the largest cape gooseberry production (Cundinamarca and Boyacá), while group E includes most accessions from southern Colombia (Nariño and Valle) and international repositories (Ecuador, New Zeeland, India and Nepal). Groups C and F combine accessions from all over Colombia, with slightly high accession numbers coming from Boyacá and Nariño in group C. Regarding the state of cultivation, groups B and C gather mostly cultivated accessions; group C contains a moderate number of weedy accessions. However, there were a high number of accessions with neither geographical origin (subpopulation C and F) nor state of cultivation (all subpopulations) information, preventing the full categorization of each subpopulation according to their passport data.

The genetic diversity estimated here as the average H_E_ per locus was relatively high within *P*. *peruviana* subpopulations, ranging from 0.35 to 0.36 (Table 4). In contrast, the F_IS_ values were low, ranging from -0.11 to -0.01 (Table 4). These results indicate an excess of heterozygosity within *P*. *peruviana* in agreement with the mixed mating system characteristic of the species. The H_O_ values ranged from 0.37 to 0.41 (Table 4). These values did not differ substantially from the H_E_ values reported, suggesting that *P*. *peruviana* could be close to a Hardy-Weinberg equilibrium due to the lack of distinct groups and high allele sharing.

**Table 4:** Average observed heterozygosity (H_O_), expected heterozygosity (H_E_) and inbreeding coefficient (F_IS_) per five *P*. *peruviana* subpopulations previously identified by ADMIXTURE.

Subpopulation	H_O_	H_E_	F_IS_
**B**	0.37	0.36	-0.01
	(0.25)*	(0.18)	(0.37)
**C**	0.38	0.35	-0.02
	(0.24)	(0.18)	(0.29)
**D**	0.41	0.35	-0.11
	(0.26)	(0.19)	(0.29)
**E**	0.39	0.35	-0.07
	(0.25)	(0.19)	(0.30)
**F**	0.38	0.35	-0.04
	(0.24)	(0.19)	(0.30)

*In parenthesis, standard deviation.

These results were further supported by the genetic differentiation between the five subpopulations detected by ADMIXTURE. The F_ST_ correlation values among subpopulations uncovered a low genetic differentiation within *P*. *peruviana*, with F_ST_ values ranging from 0.01 to 0.05 (Table 5), revealing a high gene flow within the species. Similar diversity and population differentiation results were also observed when the *P*. *peruviana* was grouped according to geographic origin (S2 and S3 Tables).

**Table 5:** Pairwise F_ST_ estimates among the five cape gooseberry subpopulations previously identified by admixture.

Subpopulation*	B	C	D	E	F
**B**	-	0.04	0.04	0.05	0.04
**C**	0.11	-	0.01	0.01	0.01
**D**	0.13	0.05	-	0.02	0.02
**E**	0.13	0.04	0.09	-	0.01
**F**	0.12	0.02	0.08	0.06	-

*Above diagonal: Average F_ST_ values per locus. Below diagonal: Standard deviation.

## Discussion

Molecular markers have allowed the detailed study of germplasm collections, revealing the history of crop domestication, discovering novel genetic diversity [42], and improving the efficiency of conventional plant breeding schemes through marker-assisted and genomic selection [43–45]. Markers such as COSIIs, IRGs, and SSRs have been used for genetic diversity studies in Colombian cape gooseberry populations, due to their highly polymorphic nature [22, 46]. However, the use of SNP markers is now more common to characterize the genetic diversity of germplasm collections, considering its abundance, reproducibility, discriminative power, and cost-effectiveness [47]. In this study, a standardized GBS pipeline was chosen to identify a new set of SNP markers, in order to evaluate a large Colombian germplasm collection of cape gooseberry.

GBS represents an innovative method for large scale SNP detection and genotyping of genetic resources [29]. In an orphan species such as cape gooseberry, molecular markers derived from high throughput sequencing technologies like GBS have allowed, in a cost-effective and time-efficient way, access to its genetic diversity and population structure [31, 32]. It has also permitted the identification of genes related to the resistance response to *Foph* [31] and quality-related traits [32] through genome-wide association studies (GWAS) in a small *P*. *peruviana* germplasm collection.

### Intermediate call SNP rates, technical replicates and passport data allowed for the selection of SNP datasets

In NGS and GBS studies, one of the most important applications is the ability to accurately and comprehensively identify genetic variation. Obtaining unbiased results usually requires a complex multi-step processing pipeline that includes pre-processing, read alignment, and variant calling. Each of these steps uses its own set of modifiable parameters, creating a significant amount of possible distinct pipelines, which vary significantly in the resulting called variants [48]. Therefore, evaluating the performance of calling methods is not straightforward and particular metrics and data sets can introduce bias into the performance test. Although previous studies in cape gooseberry obtained a high number of markers [31, 32], there is a need to optimize the SNP calling pipeline by increasing population size, as well as ensuring technical replicates to test for appropriate parameter algorithms. Here, we proposed the use of the available *P*. *peruviana* transcriptome assembly [49], for reads alignment, due to the absence of a high-quality genome for this species. Furthermore, we leveraged the common tags (64 bp reads) among well annotated genomes from the *Solanaceae* genus (*S*. *lycopersicum* and *S*. *tuberosum*) and *P*. *peruviana* to reduce the bias caused by the large amount of repetitive sequences, structural variations, and complex polyploid genomes present in plants [50]. In particular, cape gooseberry represents a mixed-ploidy genome as has been shown by classic cytogenetic analyses [2, 51]. In addition, the use of intermediate stringent parameters (mnLCov: 0.5 and mnScov: 0.7) was useful to decrease the missing data and the underestimation of diversity because of the presence-absence variations [52, 53]. As a consequence, the selected intermediate strict parameters had a reliable effect on population clustering, as was verified by the grouping of the 95 technical replicates with high genetic distance congruence (70%).

These parameters were selected based upon their ability to call SNPs in terms of read and taxon coverage, using additional values below and above to those reported in previous studies for cape gooseberry [31, 32]. Similarly, given the nature of the germplasm collection, in which related taxa were also included, the use of the aforementioned parameters and stringent mnMAF value (5%), could contribute to reduced false SNP calling and error rates, as well as homozygosity overestimation.

Moreover, the use of reference genomes to identify additional sources of diversity that went undetected when using a single reference, as has been previously used in similar species like tomato [54], was an advantage in this study. Considering the lack of reference genome in cape gooseberry, the strategy of combining related reference genomes and the use of common tags, based on the well-known synteny between the Solanaceae species (Table 1) [55], has been valuable.

### The standardized GBS pipeline allowed to differentiate the *Physalis* species in the germplasm under study

The NJ tree and PCA analyses enabled the identification of two main groups, containing *P*. *peruviana* and related taxa, as reported in previous studies [22, 31]. However, unlike those studies, the NJ tree and PCA allowed the differentiation of the related taxa into two subgroups, separating *P*. *philadelphica* and *P*. *ixocarpa* accessions from the others (Figs 2 and 3).

The *Physalis* genus, a member of the plant family Solanaceae, includes more than 90 commercial and ornamental species with high morphological diversity [56]. For this reason, different morphological and molecular studies have been carried out in order to resolve the relationships between the *Physalis* species. In concordance with the present study, Hu *et al*. [57] reported a closer phylogenetic relationship between *P*. *floridana* and *P*. *peruviana* than between *P*. *peruviana* with *P*. *philadelphica* using chloroplast markers. Similarly, a recent study that used complete chloroplast genomes conducted by Fen *et al*. [58] identified a close similarity between *P*. *angulata* and *P*. *peruviana* genomes. Both results were found in the NJ and PCA analyses reported in this study (Figs 2 and 3). Finally, the results of this study are further supported by the research conducted by Beest *et al*. [59]. Based on 22 morphological traits, it was found that *P*. *philadelphica* was close to *P*. *ixocarpa*, clustering apart from *P*. *pruinose* and *P*. *viscosa*, which were grouped with *P*. *peruviana*.

In particular, *P*. *peruviana* and *P*. *philadelphica* are considered the species with the most significant advances in their cultivation within the *Physalis* genus [26, 60]. However, the former has a center of diversification in the Andean Mountains of South America [61, 62], while the latter has a center of diversification located in North America and Central America [56, 60, 63], which is supported by this study where the two species were separated into different clusters. Overall, the results of this study demonstrated the usefulness of the common tags-derived SNPs to assess the genetic relationships in the *Physalis* genus.

The PCA revealed a grouping pattern in which most cape gooseberry cultivated accessions clustered together. However, the NJ tree uncovered additional subgroups (subpopulations B-F) within *P*. *peruviana*. Three *P*. *peruviana* accessions grouped with the related taxa subgroup A2, in both PCA and NJ analyses. These accessions are from the Nariño (09U207) and Tolima (09U291) departments of Colombia, and Poland (09U289). In another study based on orthologous genes Wei *et al*. [23], found that three *P*. *peruviana* accessions cluster together with related taxa (such as *P*. *philadelphica* and *P*. *angulata*). One of these accessions corresponds to the 09U289 (PI28570597GI) from Poland, supporting the grouping pattern obtained in this study. Additionally, one possible explanation of finding *P*. *peruviana* accessions grouping together with related taxa could be associated to misclassifications within the cape gooseberry germplasm collection as previously reported [22, 31]. For this reason, the above-mentioned accessions were removed for the *P*. *peruviana* population structure and diversity analyses.

### Cape gooseberry exhibits high genetic diversity with low population differentiation

The population structure analyses using ADMIXTURE did not show a clear separation among *P*. *peruviana* accessions neither by geographical origin nor by state of cultivation, as observed in other studies [22, 31, 32]. Likewise, the H_E_ values indicates a high diversity within this species, possibly as the sum of multiple factors including mixed ploidy, heterozygosity, mating system, and marker informativeness. The H_E_ values in this study agree with those found for the same species using SNP, SSR and COSII markers [23, 26]. However, depending on the molecular marker nature, marker number, and population size, different genetic diversity values have been reported for *P*. *peruviana* germplasm collections. For instance, low H_E_ values (0.22–0.25) have been uncovered in a small cape gooseberry population using random amplified polymorphic DNA and SSRs markers [20, 26], in which the dominant nature and population size, respectively, could underestimate the real genetic diversity. Furthermore, the seed movement of cultivated material between the different production regions may contribute to obtaining low F_ST_ values (0.01 to 0.05, Table 5) in this study, leading consequently to high homogenous but heterozygous cape gooseberry materials across production regions as revealed by the F_IS_ and F_ST_ values (Tables 4 and 5) and the H_O_/H_E_ values. Based on the diversity and genetic differentiation analyses using subpopulations derived from ADMIXTURE as well as geographical origin (S2 and S3 Tables), we obtained very similar findings, regardless of the clustering strategy. These results provide evidence of high allele sharing amongst subpopulations given the similar allele frequencies. Therefore, we inferred that the *P*. *peruviana* population could be behaving close to HWE, in accordance with its outcrossing nature and the fact that it has not undergone a domestication process [2, 3].

### Implications for cape gooseberry conservation and breeding

Characterizing plant genetic diversity is an important challenge, considering that diversity is a source of novel allele combinations that can be crucial for addressing climate and health challenges, ensuring food security, and improving nutrition of future generations. As a fruit crop, cape gooseberry possesses a remarkable gene pool that could be leveraged for germplasm conservation, human nutrition, and biotechnology applications. In this study, we characterize a large primary *P*. *peruviana* and related *Physalis* species germplasm collection that potentially represent secondary or tertiary gene pools that could increase the genetic variability needed to address future challenges for cape gooseberry production and value-chain.

As a member of the Solanaceae family, *P*. *peruviana* was proven in this study to be a representative example of the high diversity present in this family. The NJ and PCA analyses discriminate a germplasm collection with two main groups (related taxa and *P*. *peruviana*, Figs 2 and 3) and within *P*. *peruviana* five different subpopulations (Fig 4) with high genetic diversity (Table 4). Similarly, the F_ST_ pairwise revealed a relatively high gene flow between *P*. *peruviana* subpopulations regardless of the grouping method (i.e. ancestry or geographical origin, Table 5, S2 and S3 Tables) as has been found in previous studies [22].

Likewise, the results support a common origin in cultivated *P*. *peruviana*, given the distribution of the accessions within the different subpopulations as revealed by the F_IS_ values (Table 4). This statement is also supported by the inability to clearly classify these accessions according to their passport data as observed in ADMIXTURE given the similar allele frequencies within the *P*. *peruviana* subpopulations.

The study also reinforces previous inferences about the transition of cape gooseberry from wild/landrace to a cultivated state by farmer selection [2, 3]. For instance, the Nariño department (with accessions grouped in subpopulation E), considered to be the entry point of cape gooseberry from its center of origin into Colombia [3], presented a mix of wild and cultivated ancestries. These unique combinations, along with the related species diversity, could contain genetic variability that can be used for breeding to increase production, quality, and tolerance against biotic or abiotic factors.

Moreover, this study has implications for proper conservation and classification of cape gooseberry germplasm banks. Thus, the SNP dataset used in the study allowed the identification of potentially misclassified accessions within the cape gooseberry (i.e, cluster A2 in Figs 2 and 3) [22, 31]. Despite the fact that some accessions have poor or missing passport data, the common tags-derived SNPs were sufficient to capture the genetic variability of the population under study. This assessment will contribute to the establishment of core collections based on the GBS-SNP pipeline approximation, for conserving the species variability and diversity for its safeguard and sustainable use.

## Conclusions

The results of this study provide a comprehensive insight into the genetic diversity and population structure of a relatively large cape gooseberry repository in Colombia. The population structure and genetic diversity of cape gooseberry was assessed employing a standardizing GBS pipeline, using sequenced genomes from closely related species, as well as transcriptomic information from the same species. Through different SNP-calling parameters, technical replicates and passport data, the selected SNP dataset enabled the separation of *P*. *peruviana* from related taxa accessions using the NJ and PCA grouping methods. High genetic diversity but low subpopulation differentiation was observed for *P*. *peruviana*. The selection of SNPs derived from common homologue regions between closely annotated related species and cape gooseberry will allow the accurate inference of gene function in future GWAS and genomic selection studies.

## Supporting information

S1 Table Summary of the accessions of the *P*. *peruviana* and related taxa collection used in this study. (XLS)

S2 Table Average observed heterozigocity (H_O_), expected heterozigocity (H_E_) and inbreeding coefficient (F_IS_) per five *P*. *peruviana* subpopulations grouped according to their geographical regions. (XLS)

S3 Table Pairwise F_ST_ estimates among the five *P*. *peruviana* subpopulations grouped according to their geographical regions. (XLS)
